# Supplementary material for: Amplitude-Resolved Single Particle Spectrophotometry: A Robust Tool for High-Throughput Size Characterization of Plasmonic Nanoparticles
Source: Nanomaterials (Basel). 2023 Aug 23;13(17):2401. doi: 10.3390/nano13172401 (PMC10490240; doi:10.3390/nano13172401)
Supplement: Supplementary file 1 [file nanomaterials-13-02401-s001.zip › nanomaterials-2517759-supplementary.pdf]

Supplementary Information

Amplitude-Resolved Single Particle Spectrophotometry: A Robust Tool for High-Throughput Size Characterization of Plasmonic Nanoparticles

Rodrigo Calvo [1,2], Valerio Pini [1,*], Andreas Thon [1], Asis Saad [1], Antonio Salvador-Matar [1], Miguel Manso Silván [2,3] and Óscar Ahumada [1]

[1] Mecwins S.A., Ronda de Poniente, 15 2°D, Tres Cantos, 28760 Madrid, Spain
[2] Departamento de Física Aplicada, Universidad Autónoma de Madrid, Campus de Cantoblanco, 28049 Madrid, Spain
[3] Centro de Microanálisis de Materiales, Universidad Autónoma de Madrid, Campus de Cantoblanco, 28049 Madrid, Spain
* Correspondence: vpini@mecwins.com; Tel.: +34-918-049-064

1. Theoretical derivation of the relationship between the size and scattering amplitude in plasmonic nanoparticles

According to the Mie theory, the scattering cross-section [1,2] of a spherical particle with the radius r in a medium with a dielectric function ϵ_m at a given wavelength λ, can be described by the following equation:

$$\sigma_{sca}=\frac{\lambda^2}{2\pi}\sum_{l=1}^{\infty}(2l+1)(|a_l|^2+|b_l|^2) \tag{1}$$

The scattering coefficients a_l and b_l are expressed by the Riccati-Bessel spherical functions $\eta_l(x)$ and $\psi_l(x)$ and their first derivatives:

$$a_l = \frac{m\psi_l(mx)\psi_l^{'}(mx)\text{-}\psi_l^{'}(mx)\ \psi_l(mx)}{m\ \psi_l(mx)\eta_l^{'}(x)\text{-}\psi_l^{'}(mx)\eta_l(x)} \tag{2}$$

$$b_l = \frac{\psi_l(mx)\psi_l^{'}(x)\text{-}m\psi_l^{'}(mx)\ \psi_l(x)}{\psi_l(mx)\eta_l^{'}(x)\text{-}m\psi_l^{'}(mx)\eta_l(x)} \tag{3}$$

where the size parameter denoted as $x={}^{2\pi r}\!/_{\lambda}$ is defined as the ratio of the nanoparticle's characteristic dimension r to the light wavelength λ, and $m=n(r)/n_m$ represents the ratio between the refractive index of the particle $n(r)$ and the refractive index of the surrounding medium n_m. For the numerical calculation of the scattering signal, we used Mie functions based on the code of Mätzler [3].

Since the optical properties of particles with sizes comparable to the free path of the conduction electrons show significant variations compared to the bulk material, the Drude model [4] was used to define the refractive index of the gold nanoparticles.

A theoretical derivation could indeed be performed to relate the scattering amplitude to the nanoparticle diameter. However, for the sake of simplicity, a numerical derivation has been chosen instead. Using the optical properties of a gold nanoparticle surrounded by glycerol and applying Equations (1)–(3), with the Drude correction, the spectrum of nanoparticles of different sizes was calculated. From a Lorentzian fit, the scattering amplitude of each spectrum was extracted for the plasmonic maximum (A), and these values were plotted as a function of the calculated nanoparticle size (d), as shown in Figure S1.

A relationship between the amplitude of the plasmonic peak and the nanoparticle diameter (d) can be fitted with a simple exponential function:

$$A \approx a(1-e^{-b\cdot d})^c \tag{4}$$

where a, b, and c are physical quantities that depend on the optical properties of the surrounding medium and the refractive index of the particle.

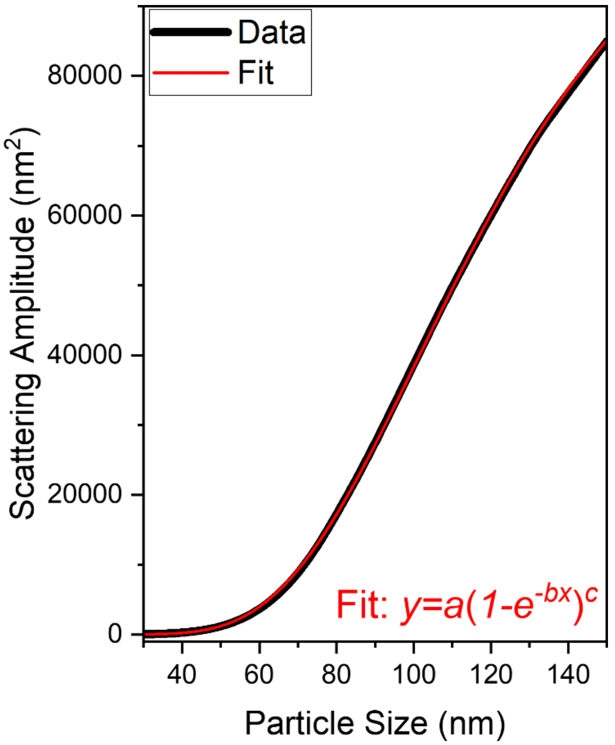

Figure S1. The black line represents the dependence of the scattering amplitude of a spherical gold nanoparticle on its diameter size. The red line is a fit performed on that same data with the formula shown in the same color in the graph; the value of R^2 for this fitting is 0.9999.

In this article, since the amplitude depends not only on the morphological and optical characteristics of the particle but also on the measurement system, a reference sample has been used as a calibration system to eliminate the influence of any instrumental dependencies by normalizing Equation (4) by A_{ref}.

Figure 2a shows that Equation (4) maintains an accurate relationship between A/A_{ref} and the nanoparticle size, even when the scattering amplitude is normalized to a reference value.

During this normalization, an interesting observation is made about the values of Equation (4). The values for b and c remain constant, but the value for a changes. As shown in Figure S2b, this value has a power dependence on the diameter of the reference batch sample (d_{ref}). This behaviour follows a specific pattern that can be modeled by fitting, i.e.,

$$a \approx \alpha d_{ref.}^{\beta} \quad (5)$$

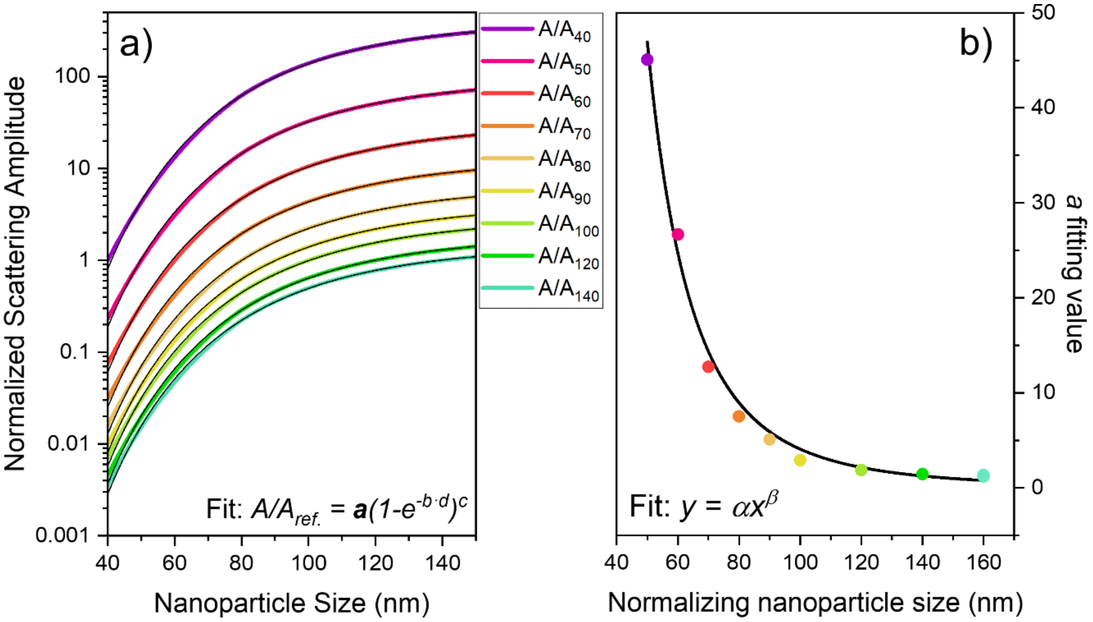

Figure S2. (**a**) Each line represents the scattering amplitude of a gold nanoparticle but normalized to the amplitude value for a given particle size established in the legend. Every plot is also fitted to Equation (4), shown in black. (**b**) The points represent the variation of variable a of Equation (4) as a function of the size of the reference nanoparticle d_{ref}. In black is shown the fitting to extract Equation (5), which has an R^2 value of ~0.988.

α and β are constants that uniquely depend on the optical properties of the system. The relationship between d and A is finally obtained by simply substituting Equation (5) into Equation (4) and isolating the nanoparticle diameter d as a function of the other variables and constants:

$$d \approx b \cdot ln\left(1 - \sqrt[c]{\frac{A/_{A_{ref.}}}{\alpha d_{ref.}^{\beta}}}\right) \qquad (6)$$

Since we considered spherical gold nanoparticles immersed in glycerol, the constants used in Equation (6) are as follows: $\alpha = 3.21 \cdot 10^7$, $\beta = -3.52$, $b = -37.85\ nm$, and $c = 14.58$. Importantly, the diameter of the reference lot must be expressed in nm to use Equation (6) correctly.

These values of α, β, b, and c have been calculated for spherical gold particles in glycerol, but in principle, this function is valid for other types of plasmonic nanoparticles and their surrounding media. Table S1 shows the values of these constants for different combinations of media and plasmonic materials, calculated using the same methodology as above.

Table S1. Different α, β, b, and c values for different surrounding and plasmonic materials for spherical nanoparticles.

**Surrounding medium**	**NP Material**	***b (nm)***	***c***	***α***	***β***
**Air (n=1)**	*Gold*	-17.57	16.17	$1.75 \cdot 10^{16}$	-6.43
	Silver	-0.033	7.73	$6.63 \cdot 10^{5}$	-2.89

**Water (n=1.33)**	*Gold*	-25.99	17.68	$2.87 \cdot 10^{13}$	-6.58
	Silver	-0.02	2.67	667.61	-1.35
**Glycerol (n=1.47)**	*Gold*	-37.85	14.58	$3.21 \cdot 10^{7}$	-3.52
	Silver	-0.0139	1.81	307.49	-1.14

2. Data Processing

The images captured by this technique show multiple nanoparticles in each image, with GNPs often forming agglomerations such as dimers, trimers, or larger clusters. To avoid the difficulty of theoretically modeling the complex spectra of nanoparticle agglomerations, they were excluded from the subsequent analysis. Prior to the spectral analysis of the sample, a color image was acquired to allow the rapid identification and classification of the monomers and their positions within the sample.

The particle detection method involves the initial identification of potential particles based on their size and shape. A grayscale test pattern is generated that takes into account the actual image acquisition parameters used and represents the typical shape of a nanoparticle. The shape of a nanoparticle is modeled by a radially symmetric two-dimensional Gaussian function. Once identified, the method stores the position and average brightness of each particle, as well as the values of the three RGB channels. Particle discrimination is achieved by analyzing the amount of scatter and the normalized values of a monochromatic component.

The classification of particles is based on two fundamental variables: the brightness of the scattering and the normalized values of a specific RGB color component; in Figure S3, the red component is used as an example. These two variables form the axes of a two-dimensional histogram that facilitates the identification of different particle types, including noise, contaminants, monomers, dimers, trimers, clusters, and more.

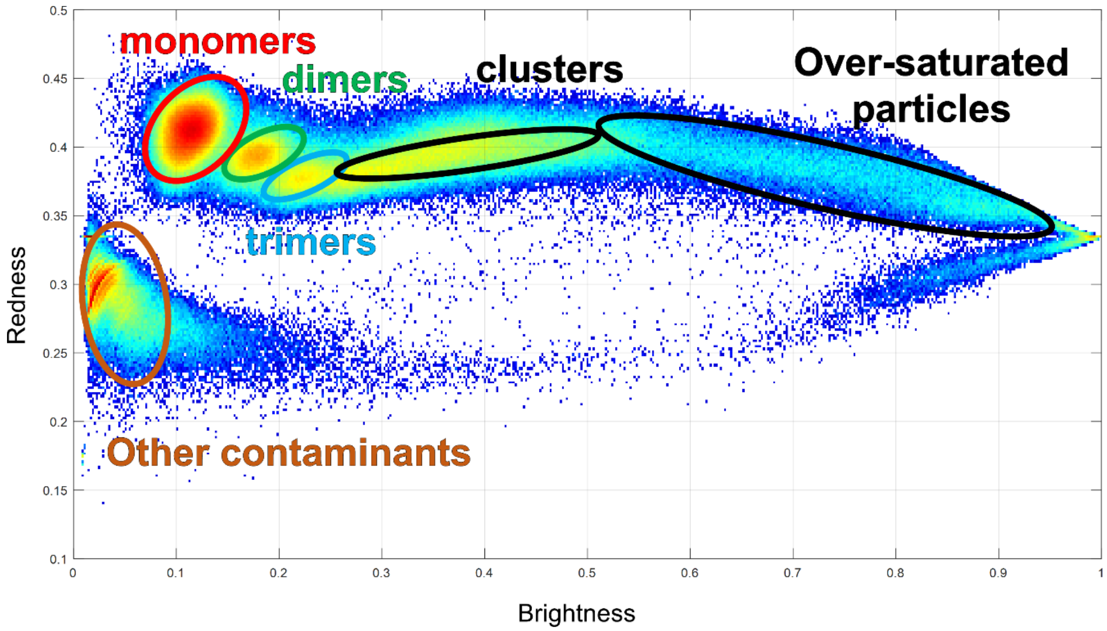

Figure S3. A color-coded two-dimensional histogram representing both the brightness and the normalized red channel values of the detected 100 nm particles. The color gradient within the diagram serves as a quantitative visual indicator: reddish values correspond to higher particle counts, while bluish colors indicate lower counts. Both the brightness and red channel data are normalized to the maximum value. This two-dimensional histogram effectively facilitates the identification and differentiation of various particle types.

Following the particle classification step, the number of particles in each class is determined and the location of each particle is recorded, providing a pair of XY coordinates for each monomer present on the surface. This information is then used to create a 3D dataset known as a spectral cube; the first two dimensions of the spectral cube correspond to the GNP coordinates, while the third dimension represents the wavelength, which is created by stacking the scattering signal at each spectral image.

Each spectral image is a 5 MP monochromatic image with an 8-bit depth, resulting in a brightness scale per pixel ranging from 0 to 255. To ensure accuracy, each monochromatic image is subjected to a pixel-by-pixel flat-field correction to eliminate variations due to the pixel-to-pixel sensitivity of the detector and distortions in the optical detection and illumination path. The following equation is used:

$$I_C = m\,\frac{I_R\text{-}DF}{I_F\text{-}DF} \tag{7}$$

In this equation, I_C is the corrected image, I_R is the raw image, DF is the dark frame, and I_F is the flat frame. The constant m is defined as the averaged value of the difference image, $I_F\text{-}DF$. A diffuse reflectance standard (SRS-99-010 from Labsphere) is used to create the flat frame.

3. Data analysis

The scattering spectra of each monomer are calculated by integrating the wavelength-dependent scattering in a circular region (about a 5 × 5 pixel area in the current setup) around each XY coordinate. A Lorentzian fit is then adjusted to each of these spectra (Figure S4):

$$S(\lambda) = S_0 + \frac{2\,\Delta}{\pi}\,\frac{w}{4\,(\lambda\text{-}\lambda_{SPS})^2 + w^2} \tag{8}$$

where $S(\lambda)$ is the wavelength-dependent scattering signal of the particle, S_0 is the baseline of the scattered emission, Δ the area under the curve, w is the width of the peak, and λ_{SPS} is the wavelength of the plasmon resonance peak.

The amplitude is therefore the value of the scattering at the resonance peak, which can be expressed as

$$A = S_0 + \frac{2\,\Delta}{w\,\pi} \tag{9}$$

Once the fitting process is complete, a filtering process based on the R-squared coefficient ($R^2 > 0.9$) is applied to discard any particles that may have been incorrectly identified and classified as monomers and that do not fit correctly to a single peak.

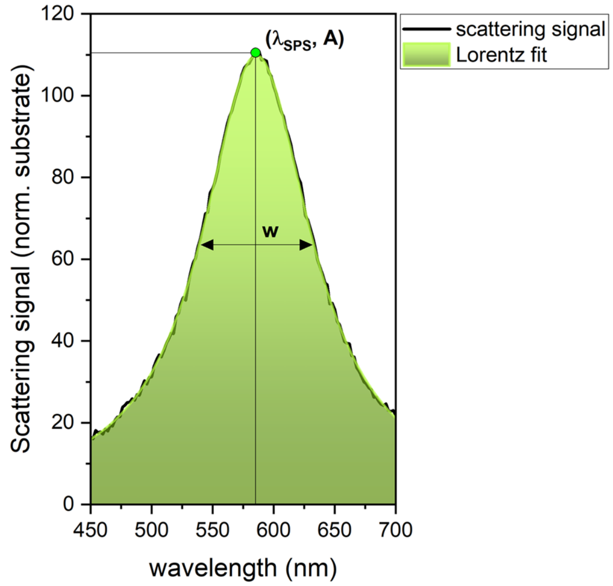

Figure S4. Scattering signal of a 100 nm single gold nanoparticle and its Lorentzian fit. For this particle, R^2 is 0.99.

4. TEM measurements and characterization

The plasmonic nanoparticles were examined by transmission electron microscopy (TEM) to determine their actual size and shape and to compare them with the nominal value specified by the manufacturer (Nanopartz). A high-voltage transmission electron microscope (JEOL JEM1010) was used to characterize the nanoparticles, with the electron beam voltage set at 100 kV for all measurements and a magnification of 300× to ensure high-resolution images. At least 500 individual nanoparticles were characterized for each batch to ensure a good statistical distribution of nanoparticle sizes and shapes.

The histogram distributions of the diameters of each nanoparticle batch are shown in Figure 5. The TEM analysis confirms that the actual size of each lot of GNPs is in very good agreement with the nominal value specified by the manufacturer, with an average deviation of about 5.7 %, as summarized in Table S2.

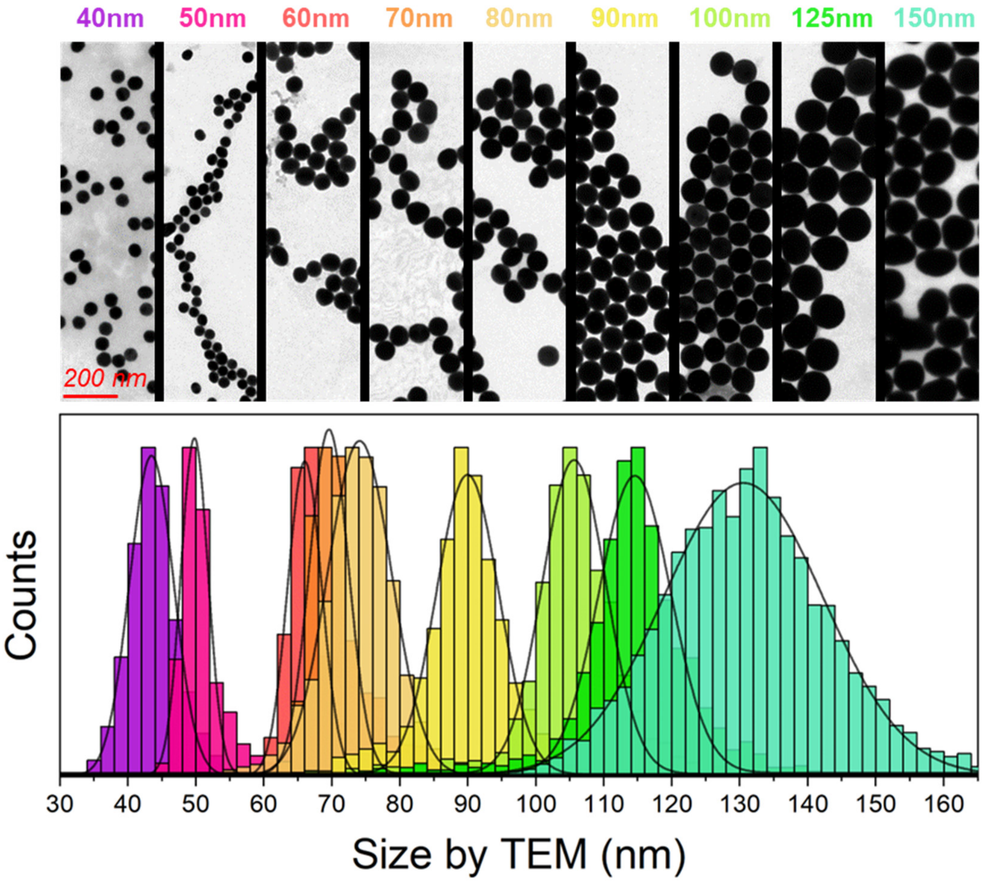

Figure S5. (**a**) TEM cropped images of the different batches of nanoparticles where several nanoparticles can be observed. The images show a good size distribution and sphericity of the particles. (**b**) Histograms of the diameters of each batch of nanoparticles; as indicated by the solid line for each batch of nanoparticles, the nanoparticles follow a Gaussian distribution with a standard deviation that is in good agreement with the nominal value given by the manufacturer.

Table S2. Nanoparticle size characterizations by TEM.

	TEM Size (nm)	Standard Dev. (nm)
**40 nm**	43.4	4.6
**50 nm**	49.7	0.6
**60 nm**	57.8	3.7
**70 nm**	69.5	0.7
**80 nm**	74.1	7.4
**90 m**	89.9	0.1
**100 nm**	103.3	3.3
**125 nm**	114.5	8.4
**150nm**	130.9	14.2

5. Size comparison between TEM and the AR-SPS method and determination of the error uncertainty

Table S3 shows the size values obtained for each batch of GNPs by both TEM and AR-SPS. The average difference between TEM and AR-SPS is 2.1 nm, while the percentage difference is less than 2.6%.

Table S3. Comparison between TEM sizes and those obtained by AR-SPS.

	TEM Size (nm)	AR-SPS Size (nm)	Discrepancy (nm)	Discrepancy (%)
**40 nm**	43.4	42.43	0.9	2.2
**50 nm**	49.7	46.83	2.8	5.8
**60 nm**	65.9	66.5	0.6	0.9
**70 nm**	69.5	69.4	0.1	0.1
**80 nm**	74.1	71.3	2.7	3.7
**90 m**	89.9	92.2	2.3	2.6
**100 nm**	103.3	106.4	3.1	3.1
**125 nm**	114.5	115.1	0.6	0.5
**150 nm**	130.5	136.4	5.9	4.5

The diameter uncertainty with AR-SPS is calculated by performing a linear propagation of Equation (6), i.e., [5]

$$\sigma_y = \sqrt{\left(\frac{\partial f}{\partial x}\right)^2 \cdot \sigma_x^2} = \left|\frac{\partial f}{\partial x}\right| \cdot \sigma_x \quad (10)$$

Once d_{ref} is determined, a, b, and c are simple constants, so the diameter uncertainty can be derived as follows:

$$\sigma_d = \left|\frac{\partial d}{\partial A_{norm}}\right| \cdot \sigma_{A_{norm}} = \left|\frac{b\sqrt[c]{A_{norm}/a}}{c\,{A_{norm}/a}\left(1-\sqrt[c]{A_{norm}/a}\right)}\right| \cdot \sigma_{A_{norm}} \quad (11)$$

where $A_{norm} = {A}/{A_{ref.}}$

6. Non-spherical particle size characterization using AR-SPS: Gold Nanorods

In the interest of elaborating on the size characterization of non-spherical particles, this section presents a brief overview of the use of spectral analysis techniques to study the aspect ratio of gold nanorods within an inhomogeneous batch. The methodology closely follows the analysis described in the primary manuscript, providing a straightforward approach to understanding particle dimensionality.

During the nanorods' synthesis, it is common to observe a change in their aspect ratio. This change typically occurs because the size of the nanoparticle remains static along one axis while it shifts along the other, effectively transitioning the nanoparticle from a

spherical to a rod-like shape [6–8]. Because such variations in the aspect ratio have a significant impact on their plasmonic properties, there is a critical need for precise metrology techniques to accurately characterize these morphological features.

Our advanced spectrophotometric technique allows us to analyze the individual spectral responses of numerous gold nanorods within a batch. We conducted an experiment on a batch containing nanorods with different aspect ratios. To illustrate our findings, we present three representative nanorods with different aspect ratios, the results of which were verified by both SEM and our novel microspectrophotometric technique, in Figure S6. As confirmed by our observations, and in agreement with the existing literature, the spectral response of nanorods with an aspect ratio greater than one exhibits two plasmon resonance peaks. These peaks correspond to plasmonic excitations along the short and long axes of the nanorod.

Although the methodology may be slightly different than for spherical nanoparticles, the size and aspect ratios of the nanorods can still be estimated by determining the amplitude and the ratio of the scattering amplitudes of the two peaks. These data can then be compared with the analytical Mie-Gans theory [9], which establishes a clear relationship between the size and aspect ratio of the nanoparticles and the amplitude and ratio of the two plasmon resonance peaks.

This is just one example of how AR-SPS can be used to address different morphological and size characterization problems. The technique needs to be adapted to the desired application, but its applicability is not limited to a specific shape.

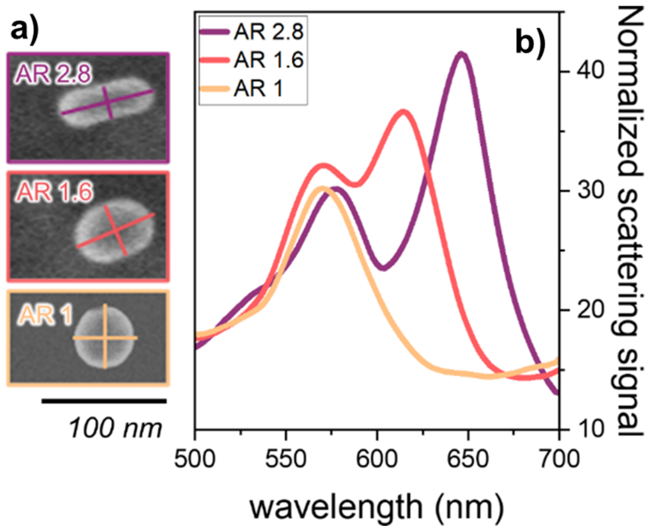

Figure S6. (**a**) SEM inset of 3 different aspect ratio (AR) gold nanorods: *2.8*, *1.6*, and *1*. (**b**) Same gold nanorod scattering spectra taken with AR-SPS. It can be observed that the shape has a strong influence on the scattering amplitude of both peaks.

7. Multiplexing with AR-SPS

The ability to multiplex is the ability to distinguish the sizes of different nanoparticles in a single sample. AR-SPS has this capability due to its ability to measure single particles.

The straightforward ability to measure the size distribution within a single batch of nanoparticles inherently demonstrates the potential to measure different sizes within a given sample. This is primarily due to the distinct advantage of our experimental technique, which is capable of measuring the individual sizes of thousands of nanoparticles deposited on a substrate by performing a spectral analysis of each individual nanoparticle.

Provided that each nanoparticle can be individually identified, there is no technical limitation to having nanoparticles of different sizes on the same substrate. Each can be

uniquely identified and spectrally distinguished. For simplicity, we performed measurements on each batch of nanoparticles separately. However, our system does not prevent us from performing a measurement on a mixture of different nanoparticles.

We demonstrated the ability to simultaneously detect the nanoparticle sizes of different batches in the experiment described below. Here, we prepared a mixture of six different nanoparticle batches (50 nm, 60 nm, 80 nm, 100 nm, 125nm, and 150 nm) and followed the same procedure described in the manuscript to measure and analyze all spectra of each individual nanoparticle.

In the dark-field image, each nanoparticle type can be individually identified (as shown by the colored circles associated with each nanoparticle type in Figure 7a). In addition, from the nanoparticle spectra of each individual particle (as shown in Figure 7b), we can easily distinguish the presence of five different nanoparticle batches in the sample.

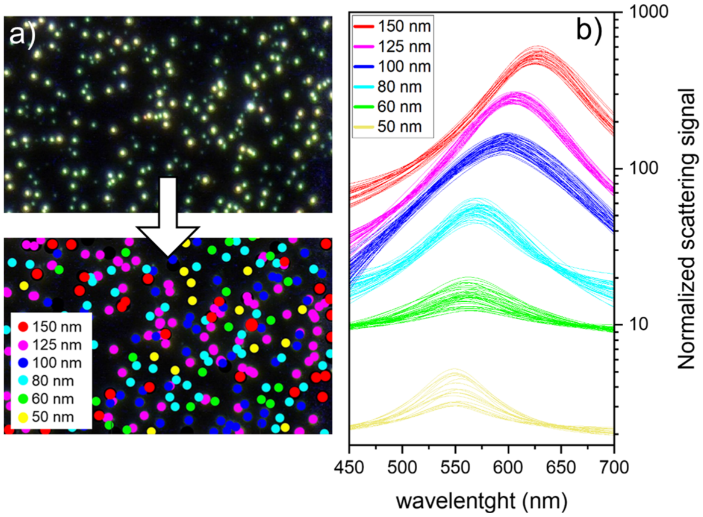

Figure S7. (**a**) Dark-field image showing all nanoparticles present from 6 batches of different sizes and their aggregates. The image below shows how the individual nanoparticle sizes can be identified according to the batch to which they belong. (**b**) Hundreds of individual spectra of each nanoparticle extracted from the multiplexing experiment.

References

1. Bohren, C. F. & Huffman, D. R. *Absorption and Scattering of Light by Small Particles*. (Wiley, 1998). doi:10.1002/9783527618156.
2. Haiss, W., Thanh, N. T. K., Aveyard, J. & Fernig, D. G. Determination of size and concentration of gold nanoparticles from UV-Vis spectra. *Anal Chem* **79**, 4215–4221 (2007).
3. Matzler, C. MATLAB functions for Mie scattering and absorption. *IAP Res Rep* **8**, (2002).
4. Kreibig, U. & Fragstein, C. v. The limitation of electron mean free path in small silver particles. *Zeitschrift für Physik* **224**, 307–323 (1969).
5. Ku, H. H. Notes on the use of propagation of error formulas. *Journal of Research of the National Bureau of Standards, Section C: Engineering and Instrumentation* **70C**, 263 (1966).
6. Feng, L. *et al.* Preparation of gold nanorods with different aspect ratio and the optical response to solution refractive index. *J Exp Nanosci* **10**, 258–267 (2015).
7. Nikoobakht, B. & El-Sayed, M. A. Preparation and Growth Mechanism of Gold Nanorods (NRs) Using Seed-Mediated Growth Method. *Chemistry of Materials* **15**, 1957–1962 (2003).

8. Tong, W., Walsh, M. J., Mulvaney, P., Etheridge, J. & Funston, A. M. Control of Symmetry Breaking Size and Aspect Ratio in Gold Nanorods: Underlying Role of Silver Nitrate. *The Journal of Physical Chemistry C* **121**, 3549–3559 (2017).

9. Yu, R., Liz-Marzán, L. M. & García de Abajo, F. J. Universal analytical modeling of plasmonic nanoparticles. *Chem Soc Rev* **46**, 6710–6724 (2017).
